# FDI-VSR: Video Super-Resolution Through Frequency-Domain Integration and Dynamic Offset Estimation

**DOI:** 10.3390/s25082402

**Published:** 2025-04-10

**Authors:** Donghun Lim, Janghoon Choi

**Affiliations:** Graduate School of Data Science, Kyungpook National University, Daegu 41566, Republic of Korea; naring@knu.ac.kr

**Keywords:** video super-resolution, dynamic offset estimation, frequency-domain integration, spatiotemporal feature extraction

## Abstract

The increasing adoption of high-resolution imaging sensors across various fields has led to a growing demand for techniques to enhance video quality. Video super-resolution (VSR) addresses this need by reconstructing high-resolution videos from lower-resolution inputs; however, directly applying single-image super-resolution (SISR) methods to video sequences neglects temporal information, resulting in inconsistent and unnatural outputs. In this paper, we propose FDI-VSR, a novel framework that integrates spatiotemporal dynamics and frequency-domain analysis into conventional SISR models without extensive modifications. We introduce two key modules: the Spatiotemporal Feature Extraction Module (STFEM), which employs dynamic offset estimation, spatial alignment, and multi-stage temporal aggregation using residual channel attention blocks (RCABs); and the Frequency–Spatial Integration Module (FSIM), which transforms deep features into the frequency domain to effectively capture global context beyond the limited receptive field of standard convolutions. Extensive experiments on the Vid4, SPMCs, REDS4, and UDM10 benchmarks, supported by detailed ablation studies, demonstrate that FDI-VSR not only surpasses conventional VSR methods but also achieves competitive results compared to recent state-of-the-art methods, with improvements of up to 0.82 dB in PSNR on the SPMCs benchmark and notable reductions in visual artifacts, all while maintaining lower computational complexity and faster inference.

## 1. Introduction

Over the past few years, there has been a growing demand for high-quality and high-resolution images and videos across diverse industries. Fields such as autonomous driving, medical imaging, and crime scene investigations rely heavily on converting low-resolution (LR) images or frames into high-resolution (HR) counterparts, underscoring the importance of super-resolution (SR) technologies. SR not only enhances resolution but also effectively reduces noise and blur, ultimately improving overall visual quality [1].

In particular, single-image super-resolution (SISR) has matured significantly, driven by breakthroughs in deep learning-based methods. From early convolutional architectures such as SRCNN [2] to more recent transformer-based models such as SwinIR [3], SISR methods have shown remarkable success in restoring fine details. However, directly applying these SISR solutions to videos typically processes each frame independently, ignoring temporal cues and leading to inconsistent or unnatural video outputs [4]. Therefore, video super-resolution (VSR) methods must address motion estimation, frame alignment, and temporal fusion [5], often requiring more sophisticated architectures than those used in SISR.

Early VSR research, such as the Bayesian adaptive method proposed by Liu and Sun [6], used optical flow [7] or LSTM-based networks [8] to capture inter-frame relationships, but these approaches still struggle with large motions and complex artifacts. Recent studies have endeavored to reduce computational overhead while improving temporal consistency in VSR. For example, Lee et al. [9] introduced a deformable convolution-based alignment network for better efficiency, while Zhu and Li [10] proposed a lightweight recurrent grouping attention network to aggregate temporal information effectively. Lu and Zhang [11] further addressed real-world degradations with a degradation-adaptive approach, highlighting the importance of handling a broad range of noise and blur conditions.

Building upon these advances, this paper addresses the following scientific questions and objectives explicitly: (1) How can we effectively integrate temporal dynamics into existing SISR architectures without extensive architectural redesigns? (2) How can we adaptively handle complex and non-uniform motion between consecutive video frames? (3) How can frequency-domain analysis improve the reconstruction of subtle textures and high-frequency details that are typically challenging for purely spatial approaches?

**Figure 1:**
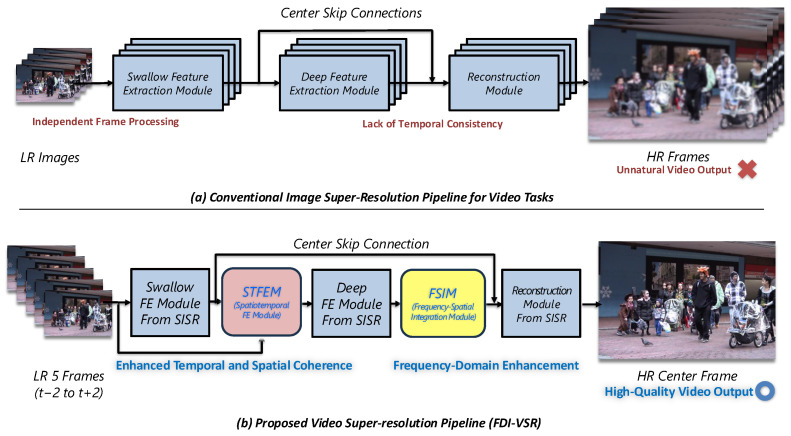
Motivation for the proposed video super-resolution framework.

To answer these questions, we propose FDI-VSR, a novel framework designed to enhance existing single-image super-resolution architectures to better address video super-resolution tasks (Figure 1). Our method specifically introduces two innovative modules: a Spatiotemporal Feature Extraction Module (STFEM), which learns adaptive offsets to handle complex and non-uniform motions between neighboring frames without relying on explicit optical flow estimation [12], and a Frequency–Spatial Integration Module (FSIM), which transforms spatial features into the frequency domain to capture global context and enhance the recovery of subtle textures and high-frequency details beyond the limited receptive field of standard convolutions [13].

The main contributions of this paper are as follows: First, we propose a flexible framework that extends conventional single-image super-resolution models to video super-resolution tasks without extensive modifications, facilitating easier adoption and deployment in practical scenarios. Second, we introduce the STFEM, which effectively manages complex motions via dynamic offset estimation and enhances temporal consistency using multi-stage temporal aggregation based on residual channel attention mechanisms [14,15]. Third, we develop the FSIM, which incorporates frequency-domain processing to complement spatial features, enabling the network to reconstruct subtle textures, periodic patterns, and low-contrast regions more effectively. Finally, extensive experiments on four widely used benchmarks (Vid4 [4], SPMCs [16], REDS4 [17], and UDM10 [18]) demonstrate that our approach achieves superior performance compared to conventional methods and competitive performance against recent state-of-the-art VSR methods while maintaining lower computational complexity.

## 2. Related Work

### 2.1. Single-Image Super-Resolution (SISR)

Single-image super-resolution (SISR) aims to reconstruct a high-resolution (HR) image from a low-resolution (LR) input by restoring lost details. Early approaches primarily employed interpolation-based methods such as bilinear or bicubic scaling, often supplemented by edge priors [19] or example-based patch matching [20]. Significant progress was later achieved with the advent of deep learning, especially convolutional neural networks (CNNs). SRCNN [2], as one of the earliest CNN-based models, demonstrated the feasibility of learning an end-to-end mapping from LR to HR domains. Subsequently, residual learning inspired by ResNet [21] led to notable improvements, exemplified by EDSR [22]. Additionally, the introduction of adversarial training, such as SRGAN [23], further enhanced the realism and perceptual quality of super-resolved images. More recently, transformer-based architectures like SwinIR [3] have set new performance benchmarks by effectively modeling local and global dependencies through self-attention mechanisms. Furthermore, HMANet [24], employing a hierarchical transformer structure combined with multi-axis attention, has demonstrated state-of-the-art performance by adaptively capturing multi-scale contextual information, thereby significantly enhancing fine-detail reconstruction capabilities.

### 2.2. Video Super-Resolution (VSR)

Video Super-Resolution (VSR) reconstructs a high-resolution (HR) central frame from multiple adjacent low-resolution (LR) frames, exploiting both spatial details and temporal correlations [5]. Unlike single-image super-resolution (SISR), VSR involves challenges like motion estimation, frame alignment, and temporal aggregation, adding significant complexity.

Early VSR methods often relied on explicit motion estimation such as optical flow [4] to warp neighboring frames for alignment. While straightforward, these approaches frequently faced difficulties in handling large motions, occlusions, or complex movements. To mitigate these limitations, frame-recurrent models employing recurrent neural networks like LSTM were proposed, propagating temporal information without explicit motion estimation [25]. However, these recurrent methods suffered from error accumulation over multiple frames, degrading quality over time.

Recently, implicit alignment techniques based on deformable convolutions have emerged, effectively managing complex and irregular motion without explicit optical flow. Models such as TDAN [5] and EDVR [26] demonstrated significant performance gains using adaptive feature sampling. Following this trend, research has further focused on lightweight and efficient architectures. Lee et al. [9] proposed deformable convolution-based networks aiming at computational efficiency, while Zhu and Li [10] introduced recurrent grouping attention for effective temporal fusion. Lu and Zhang [11] emphasized robustness against real-world degradation scenarios, highlighting the practical applicability of modern VSR methods.

Despite these advancements, current VSR techniques still face limitations, particularly in effectively aggregating spatial details across frames and fully utilizing frequency-domain information. Approaches employing single-image super-resolution (SISR) architectures frame by frame simplify development but often fail to recover subtle textures and high-frequency details, especially in low-contrast areas where frequency-domain insights are crucial.

Thus, there remains considerable room for improvement in VSR methods, specifically in effectively integrating spatial–temporal dynamics and leveraging frequency-domain analysis for superior reconstruction quality.

## 3. Proposed Method

**Figure 2:**
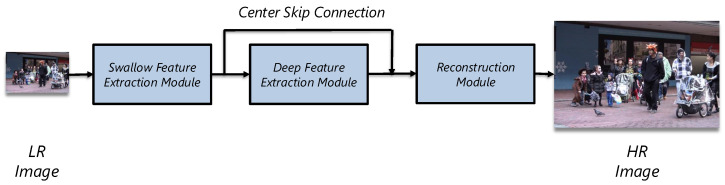
A general architecture of single-image super-resolution (SISR) models, comprising shallow feature extraction, deep feature extraction, and reconstruction stages.

To introduce our VSR approach, we first describe a typical single-image super-resolution (SISR) pipeline, which generally comprises three core stages, as illustrated in Figure 2.

A standard SISR architecture initiates with shallow feature extraction, capturing fundamental low-level features such as edges, lines, and basic textures. It then proceeds to **deep feature extraction**, where complex, hierarchical patterns are learned through deeper convolutional or transformer-based layers. Finally, the **reconstruction** stage maps these rich features back to the high-resolution (HR) image space through upsampling and refinement.

Despite recent advancements, explicitly designed video super-resolution (VSR) methods often demand extensive architectural modifications and computational resources to effectively integrate spatial and temporal information. Conversely, directly applying SISR models frame by frame simplifies development but inadequately leverages inter-frame spatial correlations and frequency-domain information, thus failing to recover subtle textures and high-frequency details, especially in low-contrast scenarios.

Motivated by these limitations, our proposed FDI-VSR framework enhances mature and computationally efficient SISR architectures by introducing two novel modules: the Spatiotemporal Feature Extraction Module (STFEM) and the Frequency–Spatial Integration Module (FSIM). The theoretical advantages of this integration are threefold:

- **Minimal architectural overhead:** By building upon mature SISR models, we avoid extensive redesign, maintaining computational efficiency and allowing easy integration into existing pipelines.
- **Effective temporal integration:** The STFEM addresses temporal misalignment through adaptive feature sampling using deformable convolutions, implicitly modeling motion without explicit optical flow estimation, thus efficiently leveraging temporal dynamics.
- **Enhanced frequency-domain awareness:** The FSIM incorporates frequency-domain analysis, explicitly capturing global structural patterns and subtle high-frequency details often overlooked by purely spatial convolutional methods, significantly improving texture and detail reconstruction, particularly in low-contrast scenarios.

Our choice to build upon mature SISR models stems from their established computational efficiency and robustness. Unlike conventional VSR methods, which often rely on complex temporal modeling such as optical flow estimation or 3D convolutions, our proposed STFEM implicitly aligns temporal features using adaptive deformable sampling, significantly reducing computational complexity. Meanwhile, FSIM explicitly incorporates frequency-domain analysis to capture global structural context overlooked by traditional convolution-based methods.

Both STFEM and FSIM modules are deliberately designed to be modular and flexible. Their modular and adaptable design enables straightforward integration into a variety of SISR architectures without requiring structural modifications. Specifically, STFEM seamlessly fits between shallow and deep feature extraction stages, and FSIM operates directly after deep feature extraction, preserving the original SISR framework’s simplicity and eliminating the need for extensive architectural redesign. These theoretical advantages and modularity underline the practical significance of our proposed approach, clearly distinguishing it from conventional VSR methodologies.

**Figure 3:**
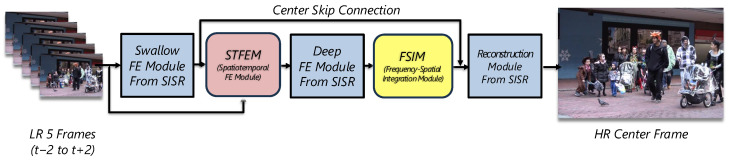
Overall structure of the proposed method. We integrate the **STFEM** and the **FSIM** into a baseline SISR model for effective video super-resolution.

As shown in Figure 3, the input is a sequence of five consecutive LR frames: $\left\{x_{t-2},x_{t-1},x_{t},x_{t+1},x_{t+2}\right\}$, where $x_{t}$ is the center frame targeted for super-resolution. Initially, shallow feature extraction processes each LR frame individually. Subsequently, these shallow features, along with the original LR frames, are fed into the STFEM, which incorporates motion information and aligns features across frames. The aggregated features from STFEM are then passed to the deep feature extractor, utilizing the inherent capability of SISR models for high-level feature learning. After deep feature extraction, features undergo global frequency analysis through the FSIM, refining them via frequency-domain characteristics. Finally, these refined features are utilized in the reconstruction stage to produce the high-resolution center frame.

The detailed architecture and operational mechanisms of STFEM and FSIM will be elaborated on in the following subsections.

### 3.1. Spatiotemporal Feature Extraction Module

**Figure 4:**
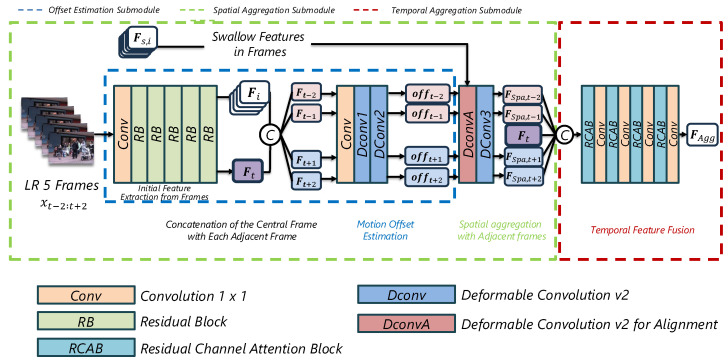
Architecture of the Spatiotemporal Feature Extraction Module (STFEM). The STFEM consists of offset estimation (blue), spatial aggregation (green), and temporal aggregation (red) submodules.

The Spatiotemporal Feature Extraction Module (STFEM) is designed to handle complex inter-frame motions by adaptively aligning and aggregating temporal information into spatial features. This module enhances the network’s ability to capture complex motions and temporal consistency through three sequential submodules: offset estimation, spatial aggregation, and temporal aggregation. Figure 4 illustrates the detailed architecture of STFEM, and the following subsections provide further explanations of each submodule.

#### 3.1.1. Offset Estimation

For each frame $x_{i}$ (where i∈{−2,−1,0,+1,+2}), shallow features $F_{i}$ are extracted:

(1) $$F_{i}=RB_{5}\left(\cdots RB_{1}\left(\mathrm{Conv}\left(x_{i}\right)\right)\cdots \right),\;F_{i}\in R^{H\times W\times C}.$$

Here, $RB_{k}$ denotes a residual block, and Conv is a standard convolution layer. The index i=0 denotes the center frame $x_{t}$.

We next estimate offsets that implicitly model motion between each neighboring frame and the center frame. For this purpose, we adopt **deformable convolution** [12,27], which adaptively adjusts sampling locations through learned offsets. This adaptive sampling enables effective alignment of features even under complex or irregular motion conditions without requiring explicit optical flow estimation. Specifically, we concatenate the shallow features of the center $F_{t}$ with those of the neighbor $F_{t,i}$ and feed them into two consecutive deformable convolution layers:

(2) $$off_{i}={\mathrm{DConv}}_{2}\left({\mathrm{DConv}}_{1}\left(\mathrm{Conv}(\mathrm{CAT}\left(F_{t,i},F_{t}\right))\right)\right).$$

**Figure 5:**
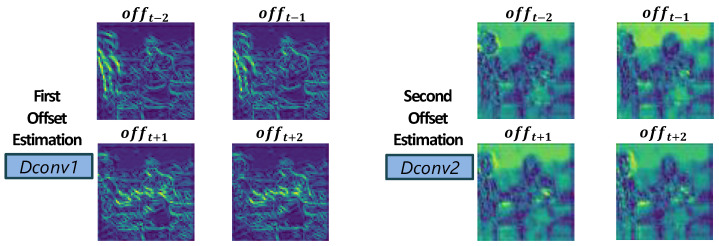
Visualization of estimated offsets between the center and adjacent frames. Green regions indicate areas of high motion.

The first deformable convolution captures coarse motion, while the second refines it further. Figure 5 visualizes these learned offsets, where regions of large or complex motion receive correspondingly larger offset values.

#### 3.1.2. Spatial Aggregation

**Figure 6:**
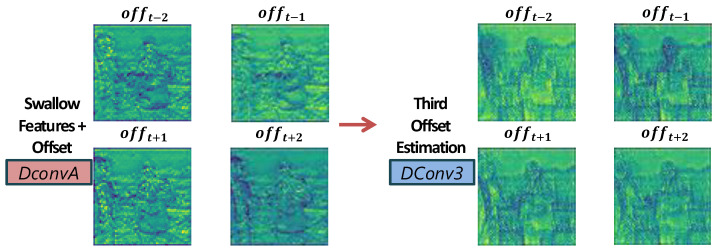
Visualization of spatial aggregation. DConv refines alignment in regions with complex motion.

Once offsets are estimated, we align the shallow features $F_{s,i}$ for each neighboring frame. Specifically,

(3) $$F_{\mathrm{Spa},i}=\mathrm{DConv}\left(\mathrm{DConvA}(F_{s,i},off_{i})\right),$$

where DConvA(·) is a deformable convolution that uses the estimated offsets $off_{i}$ to spatially align the neighboring frame’s features with the center frame. A subsequent convolutional layer refines this alignment, ensuring that objects and edges from different frames are well matched. Figure 6 illustrates how the spatial aggregation step refines the alignment in areas of complex motion.

#### 3.1.3. Temporal Aggregation

After spatial alignment, the aligned features from all frames, $\left\{F_{\mathrm{Spa},t-2,t-1,t+1,t+2},F_{t}\right\}$, are concatenated and fused via repeated residual channel attention blocks (RCABs) [14,15]:

(4) $$R_{C}\left(X\right)=\mathrm{Conv}\left(\mathrm{RCAB}(X)\right),$$

(5) $$F_{\mathrm{Agg}}=R_{C}\left(R_{C}\left(R_{C}\left(R_{C}\left(\mathrm{CAT}(F_{\mathrm{Spa},t-2,t-1,t+1,t+2},F_{t})\right)\right)\right)\right).$$

The RCABs adaptively re-weight channels to emphasize important spatiotemporal features. Intermediate 1×1 convolutions can reduce or restore channel dimensions, controlling memory usage while preserving essential information. The fused output,

(6) $$F_{\mathrm{Deep}}=FE_{\mathrm{Deep}}\left(F_{\mathrm{Agg}}\right),$$

is then fed into the deep feature extractor ($FE_{\mathrm{Deep}}$) from the baseline SISR network. This effectively injects video-specific cues into the subsequent SR layers with minimal architectural overhead.

### 3.2. Frequency–Spatial Integration Module

**Figure 7:**
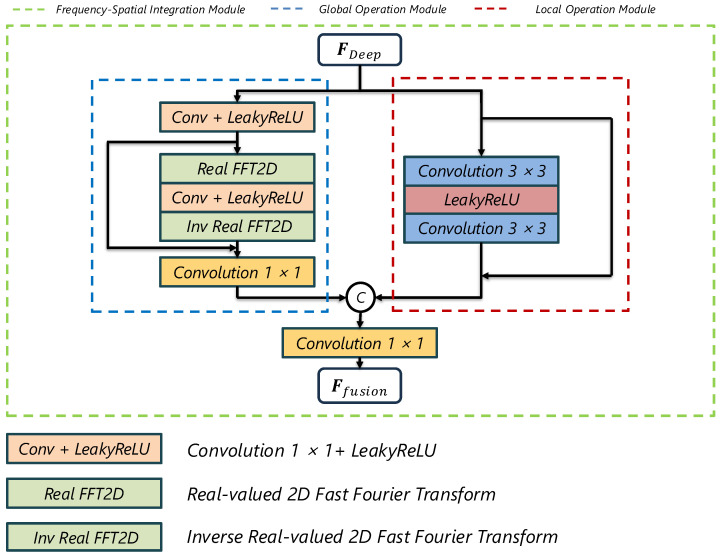
Architecture of the Frequency–Spatial Integration Module (FSIM). The FSIM consists of local operation (red), global operation (blue), and fusion (green) submodules.

While the Spatiotemporal Feature Extraction Module addresses motion and temporal consistency, convolutions alone still have a limited receptive field for capturing global context. We therefore introduce the Frequency–Spatial Integration Module (FSIM) that processes features in the frequency domain, complementing local convolutions with more global information [13]. The FSIM transforms spatial features into frequency representations, which inherently encode global structural patterns and periodic textures often missed by spatial convolutions. Figure 7 illustrates the detailed architecture of FSIM.

#### 3.2.1. Local Operation

We first apply a residual-style local convolution to refine spatial details:

(7) $$X_{\mathrm{spatial}}={\mathrm{Conv}}_{2}\left(\mathrm{LReLU}\left({\mathrm{Conv}}_{1}\left(F_{\mathrm{Deep}}\right)\right)\right)\;+\;F_{\mathrm{Deep}},$$

where LReLU is the LeakyReLU activation function [28]. This captures fine textures and edges locally, while preserving information from $F_{\mathrm{Deep}}$.

#### 3.2.2. Global Operation (Frequency Domain)

To capture global structural information and subtle high-frequency details typically lost in low-resolution images, we integrate frequency-domain analysis using the **2D Fast Fourier Transform (FFT)** [13,29,30]. Working in the frequency domain complements local spatial processing, enabling more effective reconstruction of fine textures and global patterns. Specifically, we transform the deep feature $F_{\mathrm{Deep}}$ into a frequency-aware feature $F_{CL}$:

(8) $$F_{CL}=\mathrm{LReLU}\left(\mathrm{Conv}(F_{\mathrm{Deep}})\right).$$

Then, we apply a real-valued 2D FFT (RFFT):

(9) $$X_{\mathrm{frequency}}={\mathrm{Conv}}_{2}\left(\mathrm{IRFFT}\left(\mathrm{LReLU}({\mathrm{Conv}}_{1}\left(\mathrm{RFFT}\left(F_{CL}\right)\right))\right)+F_{CL}\right).$$

Working in the frequency domain allows the module to capture large-scale structure and handle both high- and low-frequency components more flexibly than purely local convolutions. After inverse RFFT, we merge the transformed features through additional convolution and residual connections.

#### 3.2.3. Feature Fusion and Reconstruction

We concatenate the local and frequency-domain features:

(10) $$F_{\mathrm{fusion}}=\mathrm{Conv}\left(\mathrm{CAT}(X_{\mathrm{spatial}},X_{\mathrm{frequency}})\right).$$

Finally, the fusion result is skip-connected with the shallow feature $F_{s,t}$ from the center frame and passed through the reconstruction layer(s) to produce the final HR output:

(11) $$HR_{t}=FE_{\mathrm{Recons}}\left(F_{\mathrm{fusion}}+F_{s,t}\right).$$

This skip connection helps preserve low-level details from the center frame, boosting reconstruction quality.

Overall, by combining local spatial operations and frequency-domain processing, the **FSIM** enriches the network’s receptive field and enhances its ability to reconstruct both fine texture and global structure. When integrated with the **STFEM**, this design offers a powerful yet lightweight extension of standard SISR architectures to handle the challenges of video super-resolution. To provide a step-by-step summary of our entire pipeline, Algorithm 1 outlines the major procedures from shallow feature extraction through final reconstruction:
 **Algorithm 1:** Proposed Video Super-Resolution Framework **Input:** A sequence of five consecutive LR frames centered at frame *t*: $\left\{x_{t+n}|n=-2,\ldots ,2\right\}$. **Output:** High-resolution center frame $HR_{t}$. // Shallow Feature Extraction  1:**for** n=−2 to 2 **do**  2:    $F_{s,t+n}={\mathrm{FE}}_{Shallow}\left(x_{t+n}\right)$  3:**end for** // STFEM  4:**for** n=−2 to 2 **do**  5:    $F_{t+n}=RB_{5}\left(\cdots \left(RB_{1}\left(\mathrm{Conv}\left(x_{t+n}\right)\right)\right)\cdots \right)$ {Equation (1)}  6:    $off_{t+n}=DConv_{2}\left(DConv_{1}\left(\mathrm{Conv}(\mathrm{CAT}\left(F_{t+n},F_{t}\right))\right)\right)$ {Equation (2)}  7:**end for**  8:**for** n=−2 to 2 **do**  9:    $F_{Spa,t+n}=DConv\left(DConvA(F_{s,t+n},off_{t+n})\right)$ {Equation (3)}10:**end for**11:Define $R_{C}\left(X\right)=\mathrm{Conv}\left(\mathrm{RCAB}(X)\right)$ for convenience {Equation (4)}12:$F_{Agg}=R_{C}\left(R_{C}\left(R_{C}\left(R_{C}\left(\mathrm{CAT}\left(F_{\mathrm{Spa},t-2},F_{\mathrm{Spa},t-1},F_{t},F_{\mathrm{Spa},t+1},F_{\mathrm{Spa},t+2}\right)\right)\right)\right)\right)$ {Equation (5)} // Deep Feature Extraction13:$F_{Deep}={\mathrm{FE}}_{Deep}\left(F_{Agg}\right)$ {Equation (6)} // FSIM14:$X_{spatial}={\mathrm{Conv}}_{2}\left(\mathrm{LReLU}({\mathrm{Conv}}_{1}\left(F_{Deep}\right))\right)+F_{Deep}$ {Equation (7)}15:$F_{CL}=\mathrm{LReLU}\left(\mathrm{Conv}(F_{Deep})\right)$ {Equation (8)}16:$X_{frequency}={\mathrm{Conv}}_{2}(\mathrm{IRFFT}\left(\mathrm{LReLU}\left({\mathrm{Conv}}_{1}\left(\mathrm{RFFT}\left(F_{CL}\right)\right))\right)\right)+F_{CL}$ {Equation (9)}17:$F_{fusion}=\mathrm{Conv}\left(\mathrm{CAT}(X_{spatial},X_{frequency})\right)$ {Equation (10)} // Reconstruction18:$HR_{t}={\mathrm{FE}}_{Recons}\left(F_{fusion}+F_{s,t}\right)$ {Equation (11)}

## 4. Experimental Results

### 4.1. Training Details

Our **FDI-VSR** model was trained on the Vimeo-90K dataset [17], which contains 64,612 seven-frame sequences at a resolution of 448 × 256. This large and diverse dataset provides a wide range of dynamic motions and scene types, making it well suited for learning spatiotemporal features required in video super-resolution tasks. For each high-resolution frame, 256 × 256 patches were randomly extracted and then downsampled to 64 × 64 via bicubic interpolation, forming corresponding low–high resolution pairs. Data augmentation through random flips and rotations was employed to enhance the model’s robustness and mitigate overfitting. Each training sample consists of five consecutive frames with a center frame with two preceding and two following frames, to adequately capture temporal context.

### 4.2. Evaluation Details

Proposed model was evaluated on four widely recognized VSR benchmarks: Vid4 [4], SPMCs [16], REDS4 [17], and UDM10 [18]. The Vid4 dataset comprises four sequences (city, walk, calendar, and foliage), each containing approximately 30 frames at a resolution of 720 × 480. Vid4 has long served as a standard benchmark in video super-resolution research due to its moderate motion and challenging textures. The SPMCs dataset includes 11 sequences with roughly 31 frames each at a resolution of 960 × 520, providing a more diverse and dynamic testing environment with pronounced motion variations. REDS4 is a subset of the REDS dataset with complex scenes and diverse motion patterns. UDM10 [18] consists of 10 different video sequences with 2K resolution frames, offering high-quality content for evaluation. Performance was measured using PSNR and SSIM computed on the Y-channel (except REDS4, which uses RGB-channel), after discarding a four-pixel border to mitigate boundary artifacts, and our approach was compared against leading VSR models.

### 4.3. Implementation Details

All models were trained using a batch size of 16. We employed the mean squared error (MSE) loss:

$$\mathrm{Loss}={∥HR-SR∥}^{2},$$

which correlates directly with improving PSNR. We used the Adam optimizer [31] with $\beta_{1}=0.9$ and $\beta_{2}=0.99$. The learning rate was initially $2\times 10^{-4}$ and halved at 300k, 500k, 650k, 700k, and 750k iterations, for a total of 800k iterations. This multi-step decay strategy balanced stable convergence with efficient training.

Implementation was performed in Python 3.8.19, PyTorch 2.1.2, and experiments were run on an NVIDIA A6000 GPU. For reference SISR architectures, we primarily used HMANet [24] as the baseline model, which is a transformer-like model integrated with multi-axis attention.

### 4.4. Quantitative Evaluation

Table 1 summarizes the quantitative performance of our **FDI-VSR** approach compared with several state-of-the-art methods at 4× upscaling across four benchmark datasets. As demonstrated, our FDI-VSR method achieves superior performance on all datasets, with 27.29 dB PSNR and 0.8230 SSIM on Vid4, 29.84 dB PSNR and 0.8597 SSIM on SPMCs, 31.11 dB PSNR and 0.8674 SSIM on REDS4, and 39.30 dB PSNR and 0.9629 SSIM on UDM10.

Notably, our method outperforms recent approaches like FDDCC-VSR [40] and L-VSR [41] across all datasets. On Vid4, our approach improves PSNR by +0.34 dB over L-VSR and +0.50 dB over FDDCC-VSR. The performance gap is even more significant on SPMCs, where our method achieves a +0.82 dB higher PSNR than L-VSR. On REDS4, our approach attains a +0.40 dB improvement over L-VSR and +0.56 dB over FDDCC-VSR.

For the UDM10 dataset [18], our method achieves state-of-the-art performance with 39.30 dB PSNR, slightly outperforming L-VSR (39.25 dB) and significantly surpassing LRGAN (37.93 dB) and earlier methods like PFNL (35.79 dB) and DBPN (35.39 dB).

These comprehensive results across multiple benchmark datasets demonstrate the effectiveness of our proposed FDI-VSR framework, which successfully integrates spatiotemporal alignment and frequency-domain integration to achieve superior video super-resolution performance.

### 4.5. Model Complexity and Inference Speed

Beyond PSNR and SSIM, evaluating computational complexity and real-time feasibility is crucial for practical VSR applications. Table 2 compares the FLOPs and inference speed (FPS) on 64×64 inputs for several VSR models, including our proposed **FDI-VSR**.

As shown in Table 2, **FDI-VSR** requires fewer FLOPs than most recent approaches and sustains a competitive 22.54 FPS on an NVIDIA A6000 GPU. This efficiency arises from our lightweight spatiotemporal alignment (STFEM) and frequency–spatial fusion (FSIM), both of which enhance performance without incurring heavy overhead. Consequently, FDI-VSR demonstrates real-time feasibility alongside its superior reconstruction quality, making it well suited for practical video enhancement scenarios.

### 4.6. Ablation Studies

Comprehensive ablation experiments were conducted to investigate the contribution of individual components within our framework. The studies examined the impact of various submodules in the STFEM, the influence of the number of residual channel attention blocks (RCABs) in temporal fusion, the benefit of substituting a standard convolution layer with our FSIM, and the difference between Deformable Convolution v1 and v2.

**Effect of STFEM Submodules:** Table 3 presents an ablation study evaluating the contributions of individual submodules in the proposed STFEM, based on the HMANet SISR backbone. The first row corresponds to the baseline model, where none of the submodules are applied. In the first variant (second row of Table 3), only the spatial aggregation submodule is activated, utilizing shallow features from neighboring frames without offset guidance, and integrating them using a 1×1 convolution. This setting provides only marginal improvement over the baseline. The second variant (third row of Table 3) introduces the offset estimation module, which leverages both the center frame and neighboring frames to guide the spatial aggregation. This results in a substantial boost in performance—e.g., on the Vid4 dataset, the PSNR increases from 25.77 dB to 26.95 dB. Finally, the complete variant (fourth row of Table 3) incorporates all submodules, including the temporal aggregation unit, channel-wise attention, and progressive channel shrinking. This variant achieves the best performance, highlighting the effectiveness of motion-adaptive alignment and attention-driven temporal feature fusion in enhancing video reconstruction quality.

These results confirm that both motion-adaptive alignment and temporal feature fusion contribute meaningfully to performance gains in video reconstruction tasks.

**Number of RCABs in Temporal Aggregation:** Table 4 details the impact of varying the number of RCAB blocks. An increase from one to four RCAB layers leads to consistent improvements in both PSNR and SSIM, suggesting that a deeper channel attention mechanism effectively fuses temporal information, although incremental gains diminish as the number of blocks increases.

The performance improvements observed with the addition of more RCAB blocks confirm the effectiveness of deeper channel attention in consolidating temporal information.

**Frequency–Spatial Integration Module (FSIM) vs. Standard Convolution:** Table 5 compares a standard convolution layer with our proposed FSIM. The FSIM provides a notable increase in PSNR and SSIM, demonstrating its ability to capture global frequency-domain features that enhance high-frequency detail recovery.

The enhancements achieved by the FSIM indicate that incorporating global frequency-domain information is critical for recovering subtle textures and ensuring structural consistency.

**Deformable Convolution Versions:** Table 6 presents a comparison between Deformable Convolution v1 and v2. The switch to v2, which features learnable modulation scalars for finer control, yields modest yet consistent improvements, particularly in challenging scenarios involving complex motion.

The improved performance observed when using DCN v2 suggests that the learnable modulation scalars enhance the alignment capability, especially in cases of non-rigid or complex motion.

### 4.7. Visualization of Temporal Flow and Frequency Features

**Figure 8:**
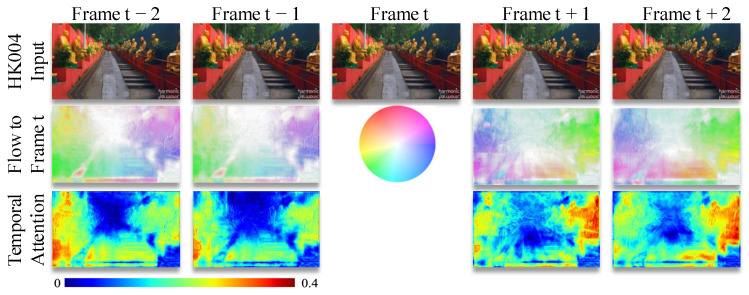
Visualization of temporal flow alignment and attention map.

To further illustrate how our proposed modules enhance VSR performance, we visualize temporal flow alignment and attention maps in Figure 8.

The circular color chart encodes motion direction and magnitude through hue and saturation, where darker tones indicate larger displacements. Offset flow fields for neighboring frames (t − 2, t − 1, t + 1, t + 2) relative to the center frame *t* demonstrate adaptive spatial alignment. Temporal attention maps reveal selective focus on regions with stable textures and edges, showing how the model prioritizes informative pixels across frames. Brighter areas in the attention maps correspond to regions where the network assigns higher importance during temporal fusion, particularly around moving objects and structural boundaries.

**Figure 9:**
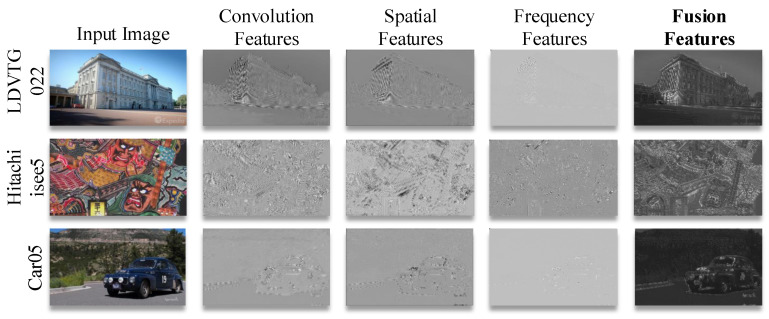
Comparison between **FSIM**-fused features and standard convolutional features.

Figure 9 compares FSIM-fused features with standard convolutional features. The FSIM’s fused features exhibit enhanced high-frequency details compared to standard convolutional features. Local spatial features preserve edge structures while frequency-domain components capture periodic patterns and global context. This synergy enables better reconstruction of both textured surfaces (e.g., brick walls) and regular geometries (e.g., window frames). The frequency-domain analysis particularly improves recovery of repeating patterns and low-contrast details that are challenging for spatial-only operations.

### 4.8. Qualitative Performance Evaluation

We compare our FDI-VSR framework against several state-of-the-art (SOTA) methods—PFNL, TDAN, D3D, and FDDCC-VSR—on a variety of challenging scenes. Overall, our approach more effectively handles complex motion and recovers subtle details such as text, thin structures, and high-frequency textures. These improvements arise from two key factors: dynamic offset estimation, which adaptively aligns frames without relying on explicit optical flow, and frequency-domain fusion, which captures global context and enhances high-frequency components. The combination of these modules allows FDI-VSR to produce sharper edges, clearer text, and fewer artifacts than competing methods.

**Figure 10:**
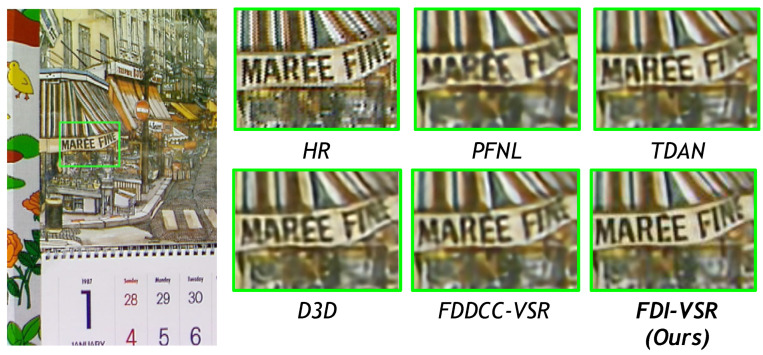
Qualitative comparison of Vid4 (Calendar) dataset for ×4 video SR.

In Figure 10, the competing methods struggle to restore the small lettering on the shop sign, leading to blurring or noise around the letters. FDI-VSR recovers more legible text and cleaner edges, indicating robust temporal alignment and effective frequency-domain enhancement.

**Figure 11:**
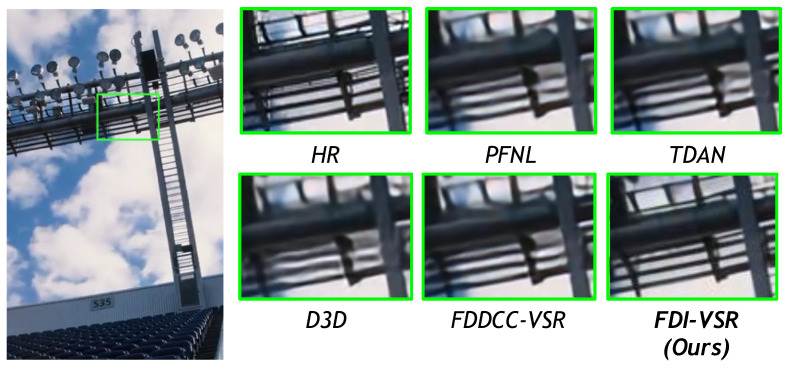
Qualitative comparison of UDM10 (002) dataset for ×4 video SR.

In Figure 11, thin metallic bars and subtle gradients pose challenges for preserving straight lines and avoiding jagged artifacts. FDI-VSR yields smoother edges and fewer distortions than PFNL or TDAN, reflecting the benefit of our dual-domain fusion in maintaining structural coherence.

**Figure 12:**
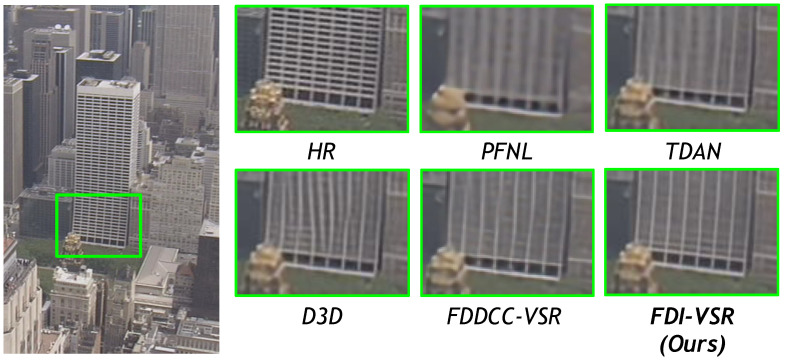
Qualitative comparison of SPMCs (HKVTG) dataset for ×4 video SR.

In Figure 12, the grid structure of the high-rise building poses alignment challenges due to high-frequency details and motion. Competing methods tend to exhibit distortion or discontinuity in the grid lines. In contrast, FDI-VSR accurately reconstructs the grid without distortion, presenting sharp and clean vertical/horizontal lines. This highlights FDI-VSR’s superior motion compensation and the effectiveness of frequency-domain fusion in preserving structural features.

**Figure 13:**
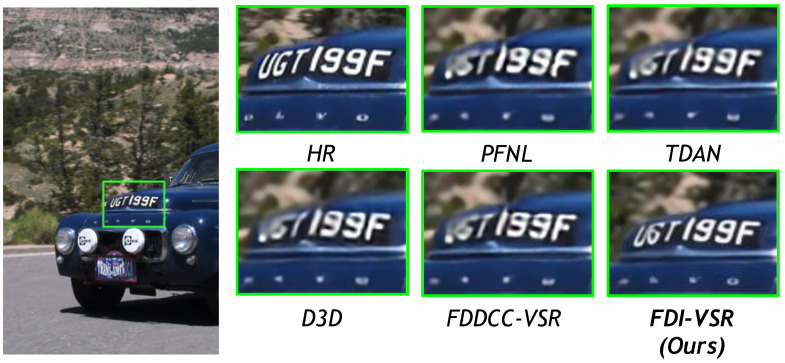
Qualitative comparison of the SPMCs (car05) dataset for ×4 video SR.

In Figure 13, license plate text demands precise reconstruction of alphanumeric characters. FDI-VSR offers visibly sharper letters than baseline methods, highlighting the synergy between accurate frame alignment and frequency-domain analysis in resolving fine details.

Overall, these visual results confirm that FDI-VSR consistently outperforms existing methods across diverse scenarios. By avoiding explicit optical flow and incorporating global frequency cues, our framework is able to reconstruct sharper textures, clearer text, and more stable temporal consistency, thus demonstrating strong practical utility in video enhancement tasks.

## 5. Conclusions

In this paper, we presented **FDI-VSR**, a novel framework for adapting single-image super-resolution (SISR) models to the video super-resolution (VSR) task by integrating two key modules: the **STFEM** and the **FSIM**. The STFEM employs dynamic offset estimation, spatial alignment, and multi-stage temporal fusion via residual channel attention blocks (RCABs) to capture complex inter-frame motion, while the FSIM leverages 2D FFT-based processing to extract global contextual information and improve the recovery of high-frequency details. Together, these components broaden the network’s receptive field and enable more accurate reconstruction of subtle textures and fine structures.

Extensive experiments on multiple benchmark datasets (Vid4, SPMCs, REDS4, and UDM10) demonstrate that our approach outperforms conventional VSR baselines such as TDAN and achieves competitive, if not state-of-the-art, performance compared to recent methods. In addition to delivering superior quantitative metrics, our framework attains these improvements with a significantly lower parameter count, thereby reducing computational complexity—a critical factor for deployment in resource-constrained environments.

Future work will focus on extending the proposed approach to Video Frame Interpolation, with the aim of developing a unified space–time video enhancement network that simultaneously addresses both spatial resolution improvement and temporal frame rate upscaling. We also plan to explore advanced attention mechanisms and refined loss functions to further boost reconstruction accuracy, particularly in challenging scenarios characterized by complex motion patterns and variable lighting conditions.

## Figures and Tables

**Table 1:** Quantitative comparison results (PSNR/SSIM) between FDI-VSR and several state-of-the-art methods on the Vid4, SPMCs, REDS4, and UDM10 datasets for a 4× scale factor. Values were calculated on the Y-channel except REDS4 (RGB-channel).

Method	Scale	Vid4 [4]		SPMCs [16]		REDS4 [17]		UDM10 [18]	
		PSNR	SSIM	PSNR	SSIM	PSNR	SSIM	PSNR	SSIM
Bicubic	×4	21.80	0.5246	25.67	0.7260	26.14	0.7292	28.47	0.8523
VESPCN (2017) [4]		25.09	0.7323	27.83	0.8230	29.29	0.8451	-	-
DBPN (2018) [32]		25.32	0.7362	27.92	0.8225	29.44	0.8475	35.39	0.9340
RCAN (2018) [15]		25.46	0.7404	28.36	0.8287	29.51	0.8489	-	-
PFNL (2019) [18]		26.40	0.8284	28.66	0.8478	29.63	0.8502	35.79	0.9374
SOF-VSR (2020) [33]		26.02	0.7713	28.21	0.8324	29.73	0.8519	-	-
ZS-Mo (2020) [34]		26.14	0.7974	28.80	0.8635	29.93	0.8634	-	-
TDAN (2020) [5]		26.16	0.7821	28.51	0.8412	29.87	0.8533	-	-
D3Dnet (2020) [35]		26.52	0.7993	28.78	0.8523	30.51	0.8657	-	-
FastDVDnet (2021) [36]		26.14	0.7719	28.53	0.8465	29.57	0.8474	-	-
TMNet (2021) [37]		26.23	0.8041	28.78	0.8640	29.91	0.8633	-	-
RSTT (2022) [38]		26.20	0.7991	28.86	0.8634	30.11	0.8613	-	-
STDAN (2023) [39]		26.28	0.8041	28.94	0.8687	29.98	0.8613	-	-
LRGAN (2023) [10]		26.80	0.8149	28.95	0.8608	29.82	0.8383	37.93	0.9575
FDDCC-VSR (2024) [40]		26.79	**0.8334**	28.87	0.8536	30.55	0.8663	-	-
L-VSR (2025) [41]		26.95	0.8188	29.02	0.8583	30.71	**0.8780**	39.25	**0.9656**
FDI-VSR (ours)		**27.29**	0.8230	**29.84**	0.8597	**31.11**	0.8674	**39.30**	0.9629

**Table 2:** Comparative analysis of model complexity and inference speed for various VSR models on 64×64 input sequences. GFLOPs indicate the number of floating-point operations (in billions) per forward pass. Note: Models marked with “^†^” are quoted directly from their respective publications and may have been measured on different hardware. The values for our FDI-VSR model were measured on an NVIDIA A6000 GPU.

Model	Input Size	Computational Cost (GFLOPs)	Inference Speed (FPS)
RBPN ^†^ [42]	64×64	247	16.3
TDAN ^†^ [5]	64×64	687	19.5
BasicVSR++ ^†^ [43]	64×64	415	30.2
VRT ^†^ [44]	64×64	409	21.1
FDI-VSR (Ours)	64×64	399	22.54

**Table 3:** Ablation study on STFEM (HMANet-based) on Vid4 and SPMCs.

Dataset	Spatial Aggregation	Offset Estimation	Temporal Aggregation	PSNR (dB)	SSIM
Vid4	✗	✗	✗	25.73	0.742
	✓	✗	✗	25.77	0.741
	✓	✓	✗	26.95	0.809
	✓	✓	✓	27.01	0.810
SPMCs	✗	✗	✗	28.15	0.807
	✓	✗	✗	28.18	0.809
	✓	✓	✗	29.07	0.843
	✓	✓	✓	29.24	0.847

**Table 4:** Effect of RCAB block repetitions.

Number of RCAB	Vid4		SPMCs	
	PSNR	SSIM	PSNR	SSIM
1	27.19	0.819	31.61	0.876
2	27.23	0.822	31.80	0.884
3	27.25	0.822	31.87	0.885
4	27.29	0.823	31.96	0.886

**Table 5:** Comparison of standard convolution vs. FSIM.

Layer Type	Vid4		SPMCs	
	PSNR	SSIM	PSNR	SSIM
Standard Conv Layer	26.89	0.809	31.13	0.870
FSIM	27.23	0.822	31.80	0.884

**Table 6:** Comparison of deformable convolution versions.

Version	Vid4		SPMCs	
	PSNR	SSIM	PSNR	SSIM
DCN v1	27.17	0.820	31.64	0.880
DCN v2	27.23	0.822	31.80	0.884

## Data Availability

This study uses publicly available datasets. The Vimeo-90K dataset used for training is available at http://toflow.csail.mit.edu/. The evaluation datasets are accessible via the following links (all links accessed and verified on 9 April 2025): Vid4 (https://people.csail.mit.edu/celiu/CVPR2011/videoSR.zip), SPMCs (https://github.com/jiangsutx/SPMC_VideoSR), REDS4 (https://seungjunnah.github.io/Datasets/reds.html), and UDM10 (https://drive.google.com/file/d/1G4V4KZZhhfzUlqHiSBBuWyqLyIOvOs0W/).
